# Complete genome sequence of the pandrug-resistant *Vibrio cholerae* strain KBR06 isolated from a cholera patient in Bangladesh

**DOI:** 10.1128/MRA.00577-23

**Published:** 2023-11-15

**Authors:** Md. Mohiuddin Kabir, Md. Rayhan Imam, Zinat Farzana, Chowdhury Faiz Hossain

**Affiliations:** 1 Department of Genetic Engineering and Biotechnology, East West University, Aftabnagar, Dhaka, Bangladesh; 2 Department of Pharmacy, East West University, Aftabnagar, Dhaka, Bangladesh; Montana State University, Bozeman, Montana, USA

**Keywords:** *Vibrio cholerae*, whole-genome sequencing, cholera, antibiotics, pandrug resistant, Dhaka, Bangladesh

## Abstract

*Vibrio cholerae* poses a serious hazard to global health and causes cholera disease in humans. Here, we present the full-genome sequence of a pandrug-resistant *V. cholerae* strain KBR06 isolated from a cholera patient in Bangladesh that exhibited intermediate resistance to only two antibiotics out of 39 among 14 antibiotic categories.

## ANNOUNCEMENT

The fast rise of numerous antibiotic-resistant strains of *Vibrio cholerae* documented in Africa, Asia, and America (1–4) has presented challenges for antibiotic therapy in recent years. Globally, the rising trend of multidrug-resistant *V. cholerae* strains linked to severe diseases is posing a substantial public health risk (5–7). Whole-genome sequencing of a pandrug-resistant (PDR) *V. cholerae* KBR06 isolated from a cholera patient has been reported here. Based on the results (Table 1), the strain was defined as a PDR according to the experts’ consensus (8).

**TABLE 1:** Antibiogram of *Vibrio cholerae* KBR06^*b*^

SL#	Antibiotic category	SL#	Antibiotics (concentration/disc)	R (mm)	IR (mm)	S (mm)	MD (mm)	SD (±)	RA
1	Aminoglycosides	1	Amikacin (30 µg)	≤14	15–16	≥17	15	1.00	IR
		2	Gentamicin (10 µg)	≤12	13–14	≥15	13	1.00	IR
		3	Netilmicin (30 µg)	≤12	13–14	≥15	9	1.53	R
		4	Kanamycin (30 µg)	≤13	14–17	≥18	11	1.15	R
		5	Streptomycin (10 µg)	≤11	12–14	≥15	9	1.00	R
2	Penicillins	6	Amoxicillin clavulanate (20/10 µg)	≤13	14–17	≥18	11	1.15	R
		7	Ampicillin (10 µg)	≤13	14–16	≥17	10	1.53	R
		8	Cloxacillin (30 µg)	≤24	–^*c*^	≥25	18	2.00	R
		9	Mecillinam (10 µg)	≤11	12–14	≥15	7	1.53	R
		10	Oxacillin (1 µg)	≤17	–	≥18	12	1.53	R
		11	Penicillin (10 units)	≤28	–	≥29	24	1.00	R
		12	Piperacillin (100 µg)	≤17	18–20	≥21	14	1.73	R
		13	Ticarcillin clavulanate (75/10 µg)	≤14	15–19	≥20	10	1.53	R
3	Macrolides	14	Azithromycin (15 µg)	≤12	–	≥13	10	1.00	R
		15	Erythromycin (15 µg)	≤13	14–22	≥23	9	2.08	R
4	Monobactams	16	Aztreonam (30 µg)	≤17	18–20	≥21	14	1.53	R
5	Cephalosporins	17	Cephalexin (30 µg)	≤14	–	≥15	11	1.00	R
		18	Cefepime (30 µg)	≤18	19–24	≥25	15	2.00	R
		19	Cefixime (5 µg)	≤15	16–18	≥19	11	1.53	R
		20	Cefotaxime (30 µg)	≤22	23–25	≥26	17	1.53	R
		21	Ceftazidime (30 µg)	≤17	18–20	≥21	14	1.00	R
		22	Ceftriaxone (30 µg)	≤19	20–22	≥23	15	1.53	R
		23	Cefuroxime (30 µg)	≤14	15–17	≥18	11	1.00	R
		24	Cephradine (25 µg)	≤12	13–17	≥18	8	1.53	R
6	Phenicols	25	Chloramphenicol (30 µg)	≤12	13–17	≥18	9	1.00	R
7	Fluoroquinolones	26	Ciprofloxacin (5 µg)	≤15	16–20	≥21	12	1.53	R
		27	Levofloxacin (5 µg)	≤16	17–20	≥21	13	1.53	R
8	Quinolones	28	Nalidixic acid (30 µg)	≤13	14–18	≥19	9	1.53	R
		29	Norfloxacin (10 µg)	≤12	13–16	≥17	9	1.53	R
9	Lipopeptides	30	Colistin^*a*^	≥4 µg/ml	≤2 µg/ml	–	5 µg/ml	–	R
		31	Polymyxin B^*a*^	≥4 µg/ml	≤2 µg/ml	–	5 µg/ml	–	R
10	Glycopeptides	32	Vancomycin (30 µg)	≤14	15–16	≥17	10	1.53	R
		33	Teicoplanin (30 µg)	≤10	11–13	≥14	7	1.53	R
11	Sulfonamides	34	Trimethoprim (5 µg)	≤10	11–15	≥16	8	1.00	R
12	Tetracyclines	35	Doxycycline (30 µg)	≤10	11–13	≥14	8	0.58	R
		36	Tetracycline (30 µg)	≤14	15–18	≥19	8	1.53	R
13	Carbapenems	37	Meropenem (10 µg)	≤15	16–18	≥19	12	1.00	R
		38	Imipenem (10 µg)	≤15	16–18	≥19	12	1.53	R
14	Nitrofurans	39	Nitrofurantoin (300 µg)	≤14	15–16	≥17	11	1.53	R
									

^*a*^ Broth dilution method.

^*b*^ The diameters of the experimentally obtained and reference clear zones have been shown in millimeters (mm); R: resistant (mm), IR: intermediate resistant (mm), S: susceptible (mm), MD: mean diameter (mm) from three independent experiments, SD: standard deviation, and RA: responses of *Vibrio cholerae* KBR06 to the antibiotics.

^*c*^ – indicates no data available in CLSI guidelines and/or not applicable.

The isolated strain grown on MacConkey agar was collected from the Dogma Hospital (Dhaka, Bangladesh). The hospital isolated it from the patient’s stool according to the CDC guidelines (9). After collection, the strain was cultured on thiosulfate-citrate-bile salt-sucrose agar plates, and yellow-colored and shiny colonies were observed which indicated the strain to be *Vibrio spp*. The strain was then enriched by culturing in alkaline-peptone-water for 8 hours at 37°C, genomic DNA was extracted using a commercial kit (Norgen Biotek, Canada), and PCR amplified its 16S rDNA using bacterial universal primers 27-forward (5′-AGAGTTTGATCCTGGCTCAG-3′) and 1,492-reverse (5′-CGGTTACCTTGTTACGACTT-3′) followed by sequencing. The obtained sequence was subjected to BLAST search at NCBI (https://blast.ncbi.nlm.nih.gov/Blast.cgi) for identification of the strain’s taxonomy. The sequence analysis results revealed that the isolate was *V. cholera*. For susceptibility testing, the disc-diffusion method was performed using antibiotic discs (Oxoid Ltd., England) on Mueller-Hinton agar plates according to the CLSI guidelines (10). For amikacin and gentamicin, the broth dilution method was used.

The Nextera XT DNA library preparation kit (Illumina) was used to construct a sequencing library from 1 ng of genomic DNA. Whole-genome sequencing (WGS) was performed on the Illumina MiSeq platform using the Illumina MiSeq v3 reagent kit to generate paired-end sequence reads. Default settings were used for all software unless otherwise indicated. FastQC v0.11.9 (11) was used for checking the sequence quality, and Trimmomatic v0.40 (https://github.com/usadellab/Trimmomatic) was used for adapter trimming. Further trimming was performed using FastP v0.36 (12). The total number of reads was 502,534, and the length of reads was ≤151. The sequencing depth was found to be ~30×, and genome coverage was ~99.9%. Unicycler v0.5.0 (adding “rotate” option) was used to assemble the sequences (13). The NCBI Prokaryotic Genome Annotation Pipeline (PGAP) v5.3 (14) was used for annotation. The number of contigs and *N_50_* values were 67 and 168.2 kb, respectively.

**Fig 1:**
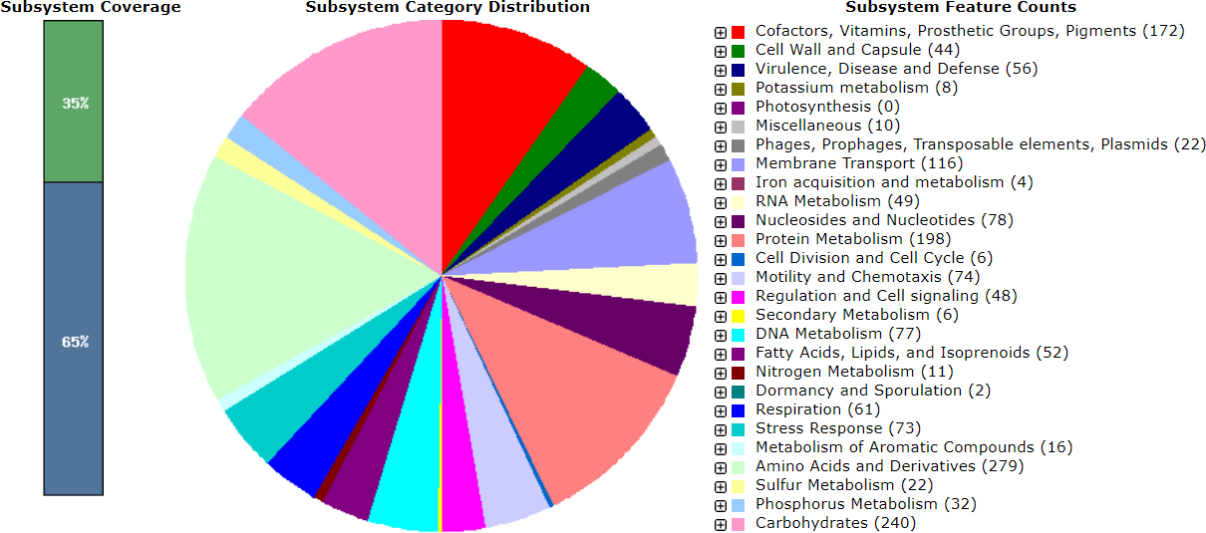
An overview of the subsystem categories assigned to the genes predicted in the genome of *Vibrio cholerae* KBR06. The metabolism of amino acids and their derivatives was consigned the highest number of genes (279 genes) followed by the metabolism of carbohydrates (240 genes) according to the subsystem category distribution of the genes assigned to various subsystems.

Annotation of the *V. cholerae* KBR06 genome identified 3,552 coding sequences. Abricate v0.8.13 (https://github.com/tseemann/abricate) and CARD (https://card.mcmaster.ca/analyze/rgi) were used to identify the antibiotic resistance (ABR) genes. The sequence assembly of *V. cholerae* KBR06 yielded a single chromosome with a genome size of 3,964,744 bp, and the GC content was 47.5%. Yamamoto et al. reported a single chromosome of *V. cholerae* and explained its reason (15). Fifty-six genes were assigned to virulence, disease, and defense (Fig. 1) according to the subsystem category distribution of the genes assigned to various subsystems based on the RAST server-based annotation (16, 17) of the *V. cholerae* KBR06 genome. Therefore, the genomic information of PDR *V. cholerae* KBR06 could be helpful to understand the genetic foundations of ABR and advance the study, management, and monitoring of ABR.

## Data Availability

The whole genome data of *V. cholerae* KBR06 have been deposited at GenBank under BioProject number PRJNA898631, accession number JAPFBX000000000, and SRA number SRR22201910. The version described in this article is version JAPFBX010000000.
